# Alterations in the aqueous humor proteome in patients with Fuchs endothelial corneal dystrophy

**Published:** 2010-11-11

**Authors:** Matthew R. Richardson, Zaneer M. Segu, Marianne O. Price, Xianyin Lai, Frank A. Witzmann, Yehia Mechref, Mervin C. Yoder, Francis W. Price

**Affiliations:** 1 Department of Pediatrics, Indiana University School of Medicine, Indianapolis, IN; 2 Department of Chemistry, Indiana University, Bloomington, IN; 3 Cornea Research Foundation of America, Indianapolis, IN; 4 Department of Cellular and Integrative Physiology, Indiana University School of Medicine, Indianapolis, IN

## Abstract

Fuchs endothelial corneal dystrophy (FECD) is a progressive disorder characterized by corneal endothelial decompensation leading to corneal edema, clouding, and vision impairment. Despite improved understanding over the last century since its first description, the exact mechanism(s) behind the pathogenesis of FECD remain unknown, and surgical correction is the only effective treatment available. Previous studies have suggested a role for changes in aqueous humor (AH) composition in FECD pathogenesis, so to explore this possibility, we probed the AH proteome for alterations correlating with end-stage corneal disease. Following albumin depletion we performed label-free quantitative tandem mass spectrometry on proteins isolated from patients with and without FECD who were scheduled to undergo routine cataract extraction. We identified 64 proteins, most of which were identified in previous AH proteomic studies of patients with cataracts, in the albumin-depleted fraction. The levels of five of these were significantly lower (afamin, complement C3, histidine-rich glycoprotein, immunoglobulin heavy [IgH], and protein family with sequence similarity 3, member C [FAM3C]), while the levels of one (suprabasin) was significantly higher in patients with FECD compared to controls (p≤0.01). We also identified 34 proteins in the albumin-bound fraction, four of which were significantly elevated in patients with FECD including a hemoglobin fragment, immunoglobulin kappa (IgK), immunoglobulin lambda (IgL), and uncharacterized protein albumin (ALB), (p≤0.01). Although it has been reported that females have a greater extent of disease than males, we were unable to detect any significant differences in protein levels due to gender. Because FECD is a progressive disorder, regression analyses were performed to determine any significant correlations with age, and of interest retinol-binding protein 3 was significantly correlated with age in patients with FECD (p≤0.01), whereas no proteins in the control group correlated with age. This is the first report indicating alterations in the AH proteome with FECD, and taken together this study suggests several novel hypotheses regarding AH proteins role in FECD pathogenesis.

## Introduction

Fuchs endothelial corneal dystrophy (FECD) was originally described by Ernst Fuchs over one hundred years ago as a disease of the corneal epithelium; since then improved technologies have enabled a more accurate description of the disease [1]. Today it is recognized as an adult–onset, progressive disorder characterized by a pleomorphic, dysfunctional, and attenuated corneal endothelium, and a thickening of Descemet’s membrane with accumulation of focal excrescences referred to as “guttae” leading to stromal edema and varying degrees of vision impairment [2] affecting at least 4% of those over 40 years of age.

It has been known for several decades that evidence of heritability is present in as many as 50% of affected patients [3], and an increased prevalence has been reported in females [1]. More recently missense mutations in genes such as collagen alpha-2(VIII) chain (*COL8A2*) have been shown to cause various forms of corneal endothelial dystrophy [4]. Moreover, much work has been invested in discovering possible mechanisms behind sporadic FECD pathogenesis. There is evidence that unfolded protein response, oxidative stress, and apoptosis play a role in the onset of the disease particularly in regard to accelerated endothelial cell loss [5-7]. Despite the great strides that have been made in understanding FECD, the exact etiology remains unclear, and currently the only permanent remedy is corneal transplantation. Thus, further investigations into possible mechanisms behind FECD that potentially could lead to novel non-invasive therapeutic approaches are warranted.

Aqueous humor supports avascular tissues in the anterior segment of the eye such as the corneal endothelium, maintains intraocular pressure, and potentially influences the pathogenesis of ocular diseases [8,9]. It has been speculated that aqueous humor (AH) composition may play a role in FECD [10,11], yet the precise role remains unknown. To investigate this possibility, a necessary first step is to determine which proteins are differentially expressed in the AH of patients with FECD. Therefore, we performed label-free quantitative mass spectrometry on AH samples from patients with late stage FECD and patients without FECD who were scheduled to undergo routine cataract extraction.

## Methods

### Sample collection

Patients were selected and samples collected as previously described [12]. Briefly, study subjects were patients scheduled to undergo routine cataract surgery at a tertiary referral center, Price Vision Group (Indianapolis, IN). Exclusion criteria were as follows: previous intraocular surgery, history of conjunctivitis or any ocular infection within the previous 3 months, intraocular inflammation, or any eye disease other than FECD. An independent review board (IRB) approved the study and all subjects signed a written Informed Consent document. Before undergoing cataract surgery, the patient's eye was anesthetized topically with proparacaine. A stab incision was made in the peripheral cornea, and 0.1 to 0.2 ml of anterior chamber fluid was aspirated using a 30-gauge needle. Aqueous humor samples were stored frozen in liquid nitrogen until analysis. A single surgeon (F.W.P.) collected all the samples. Any sample suspected of being contaminated with blood or iris pigment was discarded. Samples from 23 subjects were analyzed (11 cataract patients and 12 patients with FECD and cataracts) with 6 females in each group. The mean ages were 64.0±10.4 years (control group) and 62.8±8.8 year (FECD group); 22 patients were Caucasian and one patient with FECD was African-American (Table 1).

**Table 1:** Patient data.

**Normal**		**FECD**	
**age**	**sex**	**age**	**sex**
52	M	58	F
73	M	61	F
76	F	68	F
72	M	54	M
70	M	81	M
73	M	63	F
63	F	69	M
43	F	48	M
60	F	58	M
66	F	72	F
56	F	59	M
-	-	62	F

### Materials

Acetonitrile and ammonium bicarbonate were purchased from Fisher Scientific (Fair Lawn, NJ). Dithiothreitol (DTT) and iodoacetamide (IAA) were obtained from Bio-Rad Laboratories (Hercules, CA). Trypsin was purchased from Promega (Madison, WI). ProteoPrep immunoaffinity depletion kit was purchased from Sigma (St. Louis, MO). The following sample preparation and mass spectrometric analyses were performed at METACyt Biochemical Analysis Center (Department of Chemistry, Indiana University, Bloomington, IN).

### Depletion and protein assay

Depletion of albumin and immunoglobulin G (IgG) was performed using ProteoPrep immunoaffinity depletion kit as described in the instruction manual with some modification. As the depletion kit is designed for plasma samples and protein contents in aqueous humor (AH) is significantly lower, preliminary studies were performed to develop a protocol for optimal AH depletion, which resulted in enhanced protein identification (data not shown). Briefly, an estimate of material to be used to deplete albumin and IgG from AH was made using a bicinchoninic acid (BCA) protein assay and quantification of albumin and IgG in AH samples relative to plasma assuming total protein content of 80 µg/µl and 75% albumin and IgG in plasma. The estimated amount of material by weight was measured from the ProteoPrep immunoaffinity column and transferred to an empty spin column, and depletion was performed as described in the instruction manual.

### Trypsin digestion

Protein samples were subjected to tryptic digestion before analysis as follows: after thermal denaturation at 95 °C for 5 min, samples were reduced through the addition of DTT to a final concentration of 5 mM and incubated at 60 °C for 45 min. Alkylation was then followed by an addition of IAA to a final concentration of 20 mM for 45 min in the dark at room temperature. A second aliquot of DTT was then added, increasing the final concentration of DTT to about 10 mM. The samples were then incubated at room temperature for 30 min to quench the alkylation reaction. Next, trypsin was added (1:30 w/w) and microwave-assisted enzymatic digestion was performed at 45 °C for 15 min at the power of 50 W using CEM Discover**®** System (CEM, Matthews, NC). Finally enzymatic digestion was quenched through the addition of 0.5 µl of neat formic acid.

### Instrumentation

Liquid chromatography tandem mass spectrometry (LC-MS/MS) analyses of the tryptic digests were performed using a Dionex 3000 Ultimate nano-LC system (Dionex, Sunnyvale, CA) interfaced to an LTQ Orbitrap hybrid mass spectrometer (Thermo Scientific, San Jose, CA). Prior to separation, a 4-µl aliquot of trypsin digestion (1 µg protein equivalent) was loaded onto a PepMap300 C18 cartridge (5 µm, 300 Å; Dionex) and eluted through the analytical column (150 mm×100 µm i.d, 200 Å pores) packed with C18 magic (Michrom Bioresources, Auburn, CA). Peptides originating from protein tryptic digests were separated using a reversed-phase gradient from 10%–55% B, 99.9% acetonitrile with 0.1% formic acid over 50 min for proteins isolated from the aqueous humor, at 500 nl/min flow rate and passed through an ADVANCE ionization source (Michrom Bioresources). The mass spectrometer was operated in an automated data-dependent mode that was switching between MS scan and CID-MS. In this mode, eluted LC products undergo an initial full-spectrum MS scan from m/z 300 to 2000 in the Orbitrap at 15,000 mass resolutions, and subsequently CID-MS (at 35% normalized collision energy) was performed in the ion trap. The precursor ion was isolated using the data-dependent acquisition mode with a 2 m/z isolation width to select automatically and sequentially five most intense ions (starting with the most intense) from the survey scan. The total cycle (6 scans) was continuously repeated for the entire LC-MS run under data-dependent conditions with dynamic exclusion set to 60 s. Performing MS scanning in the Orbitrap offers high mass accuracy and accurate charge state assignment of the selected precursor ions.

### Protein identification and label-free quantitation

The acquired data were searched against the International Protein Index (IPI) human database (ipi.HUMAN.v3.69.fasta) using SEQUEST (v. Twenty-eight rev. 12) algorithms in Bioworks (v. 3.3). General parameters were set to: peptide tolerance 2.0 amu, fragment ion tolerance 1.0 amu, enzyme limits set as “fully enzymatic – cleaves at both ends,” and missed cleavage sites set at 2. The searched peptides and proteins were validated by PeptideProphet [13] and ProteinProphet [14] in the Trans-Proteomic Pipeline (TPP, v. 3.3.0). Only proteins with probability ≥0.9000 and peptides with probability ≥0.8000 were reported.

Protein quantification was performed using an in-house software package, IdentiQuantXL^TM^. The retention time of peptide for its intensity extraction was performed with an experiment-based algorithm RetentionTimeXL^TM^ .The intensity of each validated peptide was extracted and the protein quantity was calculated from peptide intensity. Student’s *t*-test was performed to determine the significance of differences between the two group means. P-values less than 0.01 were considered to be statistically significant. Linear regression analyses were performed using Spotfire^®^ (version 9.1.2).

## Results

Seeking to discover novel insights into FECD pathogenesis, we performed label-free quantitative mass spectrometry on AH samples derived from patients with and without FECD. AH samples were depleted of interfering abundant proteins such as albumin and proteins bound to albumin were eluted in a separate fraction before LC-MSMS was used for protein identification and quantification. Using stringent criteria for protein identification we identified 64 proteins in the albumin-depleted fraction (Table 2) and 34 proteins in the albumin-bound fraction (Table 3) with high confidence. There were 6 statistically significant differentially expressed proteins in the albumin-depleted fraction and 4 in the albumin-bound fraction (p≤0.01); these are listed in bold in Table 2 and Table 3. The percent of the protein sequence covered by the peptides identified with high confidence is listed for each protein along with the number of unique sequences, the coefficient of variation (CV), and the fold change compared to the protein level in patients with FECD.

**Table 2:** Proteins with altered levels identified with LC-MS/MS in the albumin-depleted fraction of AH of patients with FECD.

**Protein ID**	**Protein common name**	**Protein coverage (%)**	**Number of unique sequences**	**CV (%) Fuchs**	**CV (%) normal**	**Fold change in FED patients**	**p value**
IPI00790473	12 kDa protein	12.3	1	132.4	65.4	1.6	0.37
IPI00789547	19 kDa protein	10.3	1	113.2	62.3	−1.2	0.58
IPI00940791	20 kDa protein	42.2	7	99.8	54.7	−1.4	0.35
IPI00793626	22 kDa protein	11.2	1	155.0	91.6	2.0	0.31
IPI00942787	42 kDa protein	27.5	12	138.0	78.7	1.1	0.80
**IPI00019943**	**Afamin**	**6.7**	**2**	**66.0**	**48.1**	**−2.2**	**0.004**
IPI00022429	Alpha-1-acid glycoprotein 1	41.3	10	113.8	90.8	−1.4	0.45
IPI00020091	Alpha-1-acid glycoprotein 2	39.8	8	115.0	82.2	−1.4	0.46
IPI00847635	Alpha-1-antichymotrypsin	23.9	8	119.8	77.9	1.1	0.76
IPI00553177	Alpha-1-antitrypsin	56.7	22	135.2	81.1	1.1	0.91
IPI00022895	Alpha-1B-glycoprotein	30.1	10	116.5	56.3	1.2	0.68
IPI00166729	alpha-2-glycoprotein 1, zinc precursor	33.2	7	100.3	68.5	−1.1	0.85
IPI00922262	Alpha-2-HS-glycoprotein	11.8	1	120.2	201.3	−6.5	0.19
IPI00478003	Alpha-2-macroglobulin	17.2	16	92.0	57.3	−1.5	0.21
IPI00032220	Angiotensinogen	19.0	6	93.1	61.3	−1.2	0.57
IPI00032179	Antithrombin-III	28.7	10	79.3	44.2	−1.5	0.15
IPI00021841	Apolipoprotein A-I	39.7	10	103.8	65.0	1.2	0.71
IPI00021854	Apolipoprotein A-II	51.0	4	164.2	107.6	1.2	0.73
IPI00304273	Apolipoprotein A-IV	6.8	2	96.2	107.0	−1.1	0.81
IPI00298828	Beta-2-glycoprotein 1	9.3	2	63.0	54.9	−1.8	0.03
IPI00004656	Beta-2-microglobulin	18.5	1	95.9	121.0	−2.3	0.16
IPI00910009	cDNA FLJ53554	6.6	1	66.0	22.5	−1.1	0.63
IPI00384508	cDNA FLJ61725	0.7	1	84.5	91.8	−1.3	0.53
IPI00017601	Ceruloplasmin	35.2	23	80.4	50.4	−1.2	0.47
IPI00002147	Chitinase-3-like protein 1	3.9	1	126.3	74.0	1.2	0.73
IPI00291262	Clusterin	12.3	4	83.7	47.4	−1.2	0.48
**IPI00942927**	**Complement C3**	**3.9**	**2**	**125.9**	**33.4**	**−2.6**	**0.002**
IPI00843913	Complement component 4A	7.4	7	109.0	73.6	1.1	0.77
IPI00887154	Complement component 4B	7.4	7	109.0	73.6	1.1	0.77
IPI00032293	Cystatin-C	19.2	3	114.2	54.0	−1.2	0.63
IPI00940990	Dickkopf-related protein 3	21.7	5	92.9	82.6	−1.1	0.78
IPI00156171	Ectonucleotide pyrophosphatase/ phosphodiesterase family member 2	2.6	1	89.7	82.6	−1.6	0.20
IPI00026199	Glutathione peroxidase 3	25.7	5	68.8	47.3	−1.1	0.64
IPI00641737	Haptoglobin	25.0	12	138.0	78.7	1.1	0.80
IPI00477597	Haptoglobin-related protein	14.4	5	129.2	98.3	1.1	0.90
IPI00022488	Hemopexin	56.3	18	128.9	82.0	1.0	0.95
**IPI00022371**	**Histidine-rich glycoprotein**	**9.7**	**3**	**65.2**	**43.2**	**−2.2**	**0.002**
IPI00431645	HP protein	15.3	3	139.5	73.5	1.3	0.60
**IPI00785084**	**IGH@ protein**	**27.5**	**8**	**83.9**	**45.8**	**−2.0**	**0.01**
IPI00784985	IGK@ protein	30.2	4	127.7	37.4	1.3	0.55
IPI00784935	IGL@ protein	12.8	2	84.0	99.0	−2.2	0.12
IPI00292150	Latent-transforming growth factor beta-binding protein 2	1.0	1	81.5	166.4	1.6	0.32
IPI00022417	Leucine-rich alpha-2-glycoprotein	9.8	2	132.3	84.1	−1.2	0.69
IPI00021000	Osteopontin	10.2	2	118.4	91.2	1.5	0.39
IPI00006114	Pigment epithelium-derived factor	45.2	14	108.2	71.2	1.2	0.59
IPI00291866	Plasma protease C1 inhibitor	7.4	3	118.9	84.0	1.5	0.37
IPI00514285	Prostaglandin D2 synthase 21 kDa	14.7	2	93.9	62.8	1.1	0.73
IPI00013179	Prostaglandin-H2 D-isomerase	36.3	4	91.1	60.9	1.0	0.93
**IPI00334282**	**Protein FAM3C**	**7.1**	**1**	**61.1**	**34.7**	**−1.8**	**0.004**
IPI00556287	Putative uncharacterized protein	18.0	2	120.7	33.8	1.0	0.98
IPI00514530	Putative uncharacterized protein ACTA1	8.3	2	131.0	82.5	−1.5	0.40
IPI00022434	Putative uncharacterized protein ALB	37.3	19	91.7	70.2	1.2	0.56
IPI00924948	Putative uncharacterized protein AZGP1	31.7	5	104.6	66.6	−1.2	0.66
IPI00930072	Putative uncharacterized protein DKFZp686E23209	9.8	4	77.4	63.7	1.1	0.71
IPI00930442	Putative uncharacterized protein DKFZp686M24218	13.7	4	79.0	83.8	−1.8	0.15
IPI00022337	Retinol-binding protein 3	1.9	1	205.7	165.7	1.0	1.00
IPI00014048	RNase pancreatic	28.9	2	128.6	95.5	−1.0	0.97
IPI00386812	RIG-like 7–1	23.4	2	121.1	39.8	1.9	0.21
IPI00022463	Serotransferrin	64.5	59	103.1	59.1	1.0	0.93
IPI00745872	Serum albumin	57.5	32	78.0	57.0	1.0	0.90
IPI00383164	SNC66 protein	19.1	6	96.2	61.5	−1.2	0.65
**IPI00373937**	**Suprabasin**	**7.3**	**1**	**80.8**	**96.2**	**3.7**	**0.01**
IPI00022432	Transthyretin	69.4	11	103.8	55.5	−1.1	0.72
IPI00555812	Vitamin D-binding protein	35.4	11	116.0	30.6	1.3	0.55

The percent protein coverage (percent of total protein sequence accounted for by peptides identified), the number of unique peptide sequences, the coefficient of variation for each group, the fold change where a positive number indicates an upregulation and a negative number indicates a down regulation in control patients compared to FECD patients, and the p-value are also reported.

**Table 3:** Proteins with altered levels identified with LC-MS/MS in the albumin-bound fraction of AH of patients with FECD.

**Protein ID**	**Protein common name**	**Protein coverage (%)**	**# of unique sequences**	**CV (%) Fuchs**	**CV (%) normal**	**Fold change in FED patients**	**p value**
IPI00940791	20 kDa protein	29.2	4	37.0	118.9	−9.7	0.32
IPI00022429	Alpha-1-acid glycoprotein 1	32.3	4	46.1	66.9	−3.7	0.20
IPI00020091	Alpha-1-acid glycoprotein 2	17.9	2	48.2	95.3	−3.2	0.34
IPI00847635	Alpha-1-antichymotrypsin	25.8	8	11.2	64.0	−2.5	0.24
IPI00553177	Alpha-1-antitrypsin	54.3	24	25.7	84.0	1.3	0.60
IPI00166729	alpha-2-glycoprotein 1, zinc precursor	13.1	3	37.9	55.1	−1.8	0.25
IPI00032220	Angiotensinogen	5.0	1	39.1	78.1	−9.4	0.19
IPI00032179	Antithrombin-III	5.4	1	58.0	37.8	−2.0	0.14
IPI00021841	Apolipoprotein A-I	66.3	21	46.2	102.8	−1.5	0.62
IPI00298828	Beta-2-glycoprotein 1	8.4	2	19.1	22.7	−1.1	0.68
IPI00017601	Ceruloplasmin	4.7	2	26.7	138.9	−6.8	0.40
IPI00291262	Clusterin	4.9	2	24.1	17.7	3.8	0.03
IPI00032293	Cystatin-C	18.5	2	16.6	106.1	−3.7	0.36
IPI00168728	FLJ00385 protein (Fragment)	13.4	4	33.8	8.5	1.6	0.19
IPI00026199	Glutathione peroxidase 3	6.2	1	39.9	82.3	1.0	0.98
**IPI00796636**	**Hemoglobin (Fragment)1**	**2.4**	**1**	**13.8**	**37.1**	**5.8**	**0.001**
IPI00410714	Hemoglobin subunit alpha	28.2	3	36.5	68.6	1.5	0.35
IPI00654755	Hemoglobin subunit beta	76.9	9	24.7	115.4	1.2	0.74
IPI00022488	Hemopexin	21.2	6	33.3	82.9	−2.9	0.30
IPI00785084	IGH@ protein	29.5	9	24.4	27.5	2.0	0.04
**IPI00784865**	**IGK@ protein**	**22.5**	**3**	**13.6**	**24.2**	**2.9**	**0.002**
**IPI00829877**	**IGL@ protein**	**23.3**	**3**	**10.4**	**22.4**	**3.1**	**0.001**
IPI00009650	Lipocalin-1	9.1	1	54.9	156.3	−4.3	0.48
IPI00006114	Pigment epithelium-derived factor	22.3	7	37.8	55.1	−1.1	0.75
IPI00013179	Prostaglandin-H2 D-isomerase	32.6	3	63.9	86.5	−1.6	0.52
**IPI00878517**	**Putative uncharacterized protein ALB**	**4.9**	**1**	**23.0**	**49.4**	**3.7**	**0.01**
IPI00399007	Putative uncharacterized protein DKFZp686I04196 (Fragment)	14.6	4	24.7	29.5	1.8	0.06
IPI00930442	Putative uncharacterized protein DKFZp686M24218	9.2	3	25.2	29.4	1.8	0.06
IPI00830047	Putative uncharacterized protein ENSP00000374858 (Fragment)	28.3	2	35.2	30.8	2.6	0.04
IPI00022463	Serotransferrin	29.9	15	22.0	80.6	−1.8	0.43
IPI00745872	Serum albumin	69.0	46	32.7	21.1	2.9	0.07
IPI00018381	Tolloid-like protein 1	2.9	1	51.0	37.9	2.1	0.18
IPI00022432	Transthyretin	73.5	13	38.2	135.1	−15.6	0.35
IPI00555812	Vitamin D-binding protein	4.6	1	72.0	16.2	−1.6	0.24

The percent protein coverage (percent of total protein sequence accounted for by peptides identified), the number of unique peptide sequences, the coefficient of variation for each group, the fold change where a positive number indicates an upregulation and a negative number indicates a down regulation in control patients compared to FECD patients, and the p-value are also reported. ANOVA revealed significant differences in females compared to males in both groups indicating potentially important ramifications for increased FECD incidence in females (p≤0.01). Albumin bound–pooled–triplicate injections; n=23.

There were no statistically significant differences between males and females in either group or among all patients included in this study using a cut off p value of 0.01. Using linear regression analyses, it was determined that there were no individual proteins whose levels were significantly associated with age in the normal group. However, at least one protein, Retinol-binding protein 3 was positively correlated with age in the FECD group (R^2^=0.49; p≤0.01). Overall, protein levels were correlated somewhat more with age in patients with FECD than in controls (R^2^=0.20 [FECD] and 0.06 [control]).

## Discussion

FECD causes progressive vision impairment and currently is a leading indication for corneal transplantation [15]. Therefore, identification of targets for non-invasive therapy that could slow or halt disease progression would benefit many patients. In this study we sought to probe the AH proteome for altered protein levels which correlate with end-stage disease and could serve as novel therapeutic targets in addition to providing insights into endothelial dysfunction leading to FECD. AH samples provided by donor patients with cataracts and patients with FECD and cataracts were analyzed using a label-free quantitative mass spectrometric approach. Protein identification was validated using Peptide- and Protein Prophet to ensure that only high confidence identifications were reported.

We identified several protein alterations in AH with FECD which may have important implications in the disease process. FECD patients exhibited a 2.2 fold decrease in afamin, a vitamin E binding plasma glycoprotein in the albumin super family. Afamin deficiency has been associated with several other chronic disorders such as atherosclerosis, ischemic heart disease, immune deficiency, certain cancers, and neurologic syndromes with an oxidative stress component [16]. Heiser et al. [17] previously reported in an experimental model that afamin was protective of cortical neuronal cells under apoptotic conditions, and a separate group reported its production by cerebrovascular endothelial cells suggesting a possible source in the AH [18]. FECD is characterized by accelerated endothelial cell loss, and several studies have shown that apoptosis is increased in the corneal endothelial cells (CEC) of patients with FECD [5,19,20]. Furthermore, the guttae, which are characteristic of FECD, initiate in the central cornea, the area most exposed to oxidative stress. Therefore, it would be interesting to investigate whether patients with FECD have significant decreases in afamin in the AH early in the disease process and whether or not that contributes to disease by loss of a protective effect on CECs. Furthermore,. Heiser et al. [17] also noted that Vitamin E and afamin synergistically enhance cell survival. So it would be interesting to investigate whether a Vitamin E deficiency in patients with FECD could be a contributing factor as well.

We also found a 2.2 fold decrease in histidine-rich glycoprotein (HRG) in the AH of patients with FECD. HRG is a serum protein previously identified in the AH and known to be involved in protective processes such as clearance of apoptotic cells [21]. A decrease in such a protective effect could contribute to disease progression in FECD patients. Furthermore, in FECD patients we observed a 2.6 fold decrease in complement C3, another protein involved in apoptotic cell clearance [22], providing another possible insight into FECD pathogenic mechanisms involving apoptosis. Complement C3 is also known to be a key inflammatory protein activated in Alzheimer disease. In a transgenic mouse model, it was shown that deficiency of complement C3 leads to accelerated amyloid beta deposition and neurodegeneration [23]. Therefore, it would be interesting to determine if reduced complement C3 levels in the AH of patients with FECD also plays a role in the FECD disease process, in which the endothelium deposits excessive amounts of basement membrane material of abnormal composition, resulting in guttae formation [24].

Regarding alterations in the albumin-bound fraction, it is interesting to note that the four proteins that were differentially expressed were upregulated by 3–6 fold in patients with FECD and are all common serum proteins. It is also interesting to note that there was a 3.8 fold upregulation of clusterin (CLU) in the albumin-bound fraction of AH in patients with FECD (p=0.03), but there was no change in the albumin-depleted fraction. CLU has been previously reported as an albumin-bound protein in human plasma [25], and it is thought to play a role in maintenance of cells at tissue-fluid interfaces, inhibition of complement mediated cell lysis, and protection from apoptosis [26]. Indeed, it was recently shown that CLU attenuated oxidative stress induced apoptosis in human CECs [27]. Jurkunas et al. [28] previously reported a 5.2 fold increase in presecretory CLU protein in CECs of patients with FECD but no change in the mature modified form for secretion [28]. Thus, it is possible that CLU is upregulated in FECD CECs as a compensatory response to cell loss due to apoptosis but the increased secreted protein is being sequestered by albumin thus preventing the intended protective effect.

FECD is a progressive disorder, so we used regression analyses to see if any particular protein was associated with age. We determined that retinol-binding protein 3 was significantly associated with age in patients with FECD whereas there were no positive correlations in the control (cataract only group). Alpha-1B-glycoprotein was also highly associated with age among patients with FECD (R^2^=0.46; p=0.015), indicating possible candidates for markers of the disease. It is also important to note that no differences were detected in AH proteins between men and women in this study suggesting that gender differences in severity of FECD are not related to the AH proteome.

### Conclusions

The past one hundred years of investigation into Fuchs endothelial corneal dystrophy (FECD) has been marked by a dramatic improvement in our understanding of the underpinnings of the disease at the tissue, cellular, and molecular level. Nevertheless, no known single causal factor has been discovered, and it now appears that it is a multifactorial disease. Consistent with this line of thought, we present here for the first time several differences in protein concentration that occur in the fluid that bathes the dysfunctional tissue, all of which may be synergistically contributing to the dysfunction. Several of these proteins may be derived from serum and thus serve as a set of markers for the disease and enable detection before a deficit in vision. These patients could then be candidates for alternative therapies that might obviate the need for surgery later in life. In future work, we will confirm these differentially expressed proteins in FECD patient AH as well as test plasma samples using alternative protein quantitation strategies such as western blotting. Also, to search for biomarkers of FECD, we will perform regression analyses seeking AH proteins whose levels correlate strongly with the extent of FECD.
